# Evaluation of an implementation facilitation strategy for settings that experience significant implementation barriers

**DOI:** 10.1186/1748-5908-10-S1-A46

**Published:** 2015-08-20

**Authors:** Mona J Ritchie, JoAnn E Kirchner, Louise E Parker, Geoffrey M Curran, John C Fortney, Jeffery A Pitcock, Laura M Bonner, Amy M Kilbourne

**Affiliations:** 1 Mental Health Quality Enhancement Research Initiative (QUERI), Department of Veterans Affairs, North Little Rock, AR, 72114, USA; 2 Department of Psychiatry, University of Arkansas for Medical Sciences, Little Rock, AR, 72114, USA; 3 Graduate School, University of Arkansas, Fayetteville, AR, 72701, USA; 4 Department of Management and Marketing, University of Massachusetts, Boston, MA, 02125, USA; 5 VA Center for Mental Healthcare & Outcomes Research, Central Arkansas Veterans Healthcare System, North Little Rock, AR, 72114, USA; 6 HSR&D Center of Innovation for Veteran-Centered and Value-Driven Care, VA Puget Sound Healthcare System, Seattle, WA, 98108, USA; 7 Department of Psychiatry and Behavioral Sciences, University of Washington, Seattle, WA, 98195-6560, USA; 8 Geriatric Research, Education and Clinical Center (GRECC), VA Puget Sound Healthcare System, Seattle, WA, 98108, USA; 9 VA Quality Enhancement Research Initiative (QUERI), VA Health Services Research and Development, Department of Veterans Affairs, Washington, DC, 20420, USA; 10 Department of Psychiatry, University of Michigan Medical School, Ann Arbor, MI, 48109, USA

## Panel overview

Though several implementation and quality improvement strategies have been shown to be effective in implementing programs and practices in routine clinical settings [1,2], little work has been done in developing and testing implementation strategies in settings that experience significant implementation barriers. This VA funded study evaluated a highly partnered implementation facilitation (IF) strategy [3] within the context of a Department of Veteran Affairs (VA) mandate for implementation of Primary Care-Mental Health Integration (PC-MHI) [4]. The IF strategy consisted of a national expert external facilitator (EF) and two internal regional facilitators (IRFs) who partnered with regional, medical center, and clinic leadership and staff in two VA regional networks to implement PC-MHI. Facilitators helped partners design/adapt their PC-MHI programs, develop site-specific implementation plans, and identify/address implementation barriers. They also identified and engaged key stakeholders at all organizational levels; conducted academic detailing, marketing, staff training, patient education, formative evaluation, and audit and feedback; assisted with technical issues; and established learning collaboratives. The EF had expertise in the evidence-base for PC-MHI and implementation activities. The IRF had protected time to support implementation activities, was embedded within the clinical organization at the regional level, and was familiar with local and regional organizational structures, procedures, culture, and clinical processes. We used a quasi-experimental, Hybrid Type III design [5] and mixed methods to test effectiveness of the IF strategy and document IF activities. National VA MH leadership has adopted this IF strategy for sites facing challenges to adopting evidence-based practices [4,6,7]. This panel presents findings from the project's three components: A quantitative study of facilitation outcomes, a qualitative study of the facilitation process and its outcomes, and a qualitative study of facilitation skill transfer.

## Quantitative outcomes of using facilitation in implementing primary care - mental health integration

The study tested the effectiveness of the implementation (IF) strategy hypothesizing that, compared to national technical assistance support alone, national support plus IF would improve implementation of PC-MHI. The RE-AIM (Reach, Effectiveness, Adoption, Implementation, and Maintenance) Framework guided testing of the IF strategy's effectiveness [8,9].

Two regions were recruited to receive the IF strategy and two matched regions were recruited for comparison. Regional MH leadership identified primary care (PC) clinics unlikely to implement PC-MHI without assistance. PC clinics in comparison regions were matched to clinics in IF regions. The sample included 14 PC clinics, 174 PC providers and 98,758 PC patients. To evaluate implementation outcomes, administrative data was extracted for two six month periods, 9-15 months and 21-27 months following completion of an implementation plan at IF clinics. Generalized estimating equations were used to control for observations clustered within sites.

For the first six month period, PC patients at clinics receiving IF were more likely to be seen by PC-MHI providers (*Reach) *(OR = 8.93, p<0.001) than patients at comparison clinics. PC providers were more likely to refer at least one patient to PC-MHI (*Adoption*) (OR = 7.12, p = 0.029) than providers at comparison clinics and a greater proportion of PC providers' patients were referred to PC-MHI (*Adoption*) (β=0.027, p<0.001) at IF clinics. There was no difference between IF and comparison clinics in the likelihood of patients being referred for a first time visit to specialty mental health care (*Effectiveness*) or the percentage of patients receiving same day access to PC-MHI (*Implementation*). Similar results occurred during the second six month period (*Maintenance*).

This study documents the effectiveness of IF compared to technical assistance in the VA PC-MHI mandate and provides an evidence-based intervention for sites unable to implement programs without additional assistance. VA MH leadership has adopted the IF strategy for sites facing challenges adopting evidence-based practices.

## Examining inside the black box of implementation facilitation: process and effects on program quality

We explored inside the IF black box to document how the process changes over time and in response to circumstances. Specifically, we conducted monthly qualitative debriefings with the facilitators and semi-structured interviews with the facilitators and clinicians and managers at clinics, affiliated medical centers and regional networks over a two and half year period. Additionally, we asked experts to rate program quality and fidelity to evidence at the IF clinics and their matched comparisons [10,11].

Based on a qualitative content analysis, we determined that although certain IF activities tended to occur predominantly during particular periods, ever evolving context dictated the presence and intensity of most activities at particular times and in particular places. We observed systematic differences between the two regions and identified both regional and facilitator characteristics that may explain these differences. We also examined what facilitators, clinicians, and managers valued most about facilitation and found systematic variation. We explored the widely held assumption that facilitation activities fall into two broad categories, "doing" for others and "enabling" others to do things for themselves [1,12-15]. We found that although some activities appear to fit exclusively into one category, most do not.

Additionally, we examined the effect of IF on clinics' ability to implement evidence-based and high quality programs. Midway through the study, seven IF but only three comparison clinics had implemented a program; experts rated IF clinic programs' quality and adherence to evidence most highly. At the end of the study, all IF but only five comparison clinics had programs. All but one IF clinic had a higher rated program than its comparison.

In summary, we found that IF can foster implementation of high quality and evidence-based practices. We also found that facilitation activities do not occur according to a defined series of stages but rather flexibly in response to local circumstances.

## Transferring implementation knowledge and skills to improve healthcare delivery systems

We explored how experts in implementation facilitation (IF) can help healthcare system change agents learn how to facilitate implementation of evidence-based programs. For two and a half years, we conducted monthly debriefing interviews with a national expert external facilitator (EF) who was mentoring and coaching two internal regional facilitators (IRFs) in facilitating implementation of a VA policy initiative for Primary Care-Mental Health Integration (PC-MHI) at eight primary care clinics. Interviews focused primarily on the EF's efforts to help the IRFs become experts in IF processes. We also conducted two semi-structured qualitative interviews with each facilitator, midway through and at the end of the intervention.

Our qualitative content analysis revealed that although the EF helped IRFs learn general implementation facilitation knowledge and skills, the EF also identified IRFs' individual strengths and weaknesses and tailored mentoring and coaching activities to their characteristics. The EF used a variety of methods to help IRFs learn IF skills, including both active methods (providing information, modeling and coaching) and participatory ones. She also used cognitive supports (making thinking visible, using heuristics, sharing IF experiences) and psychosocial supports, as well strategies to promote self-learning. Additionally, the EF tailored the process to sites' implementation needs. Over time, the EF pulled back from IRFs, increasingly turning responsibility for IF activities over to them. IRFs responded differently to this process with one IRF independently "breaking away" and the other being "pushed out of the nest." In addition to helping IRFs learn the skills they needed for facilitating PC-MHI implementation, the EF helped IRFs to identify and modify interpersonal styles that could hinder success of facilitation efforts.

This study addresses the critical but understudied area of how implementation scientists can transfer facilitation skills that incorporate evidence-based implementation interventions and strategies to internal change agents to help healthcare organizations implement effective programs.
